# Evidence for Brainstem Contributions to Autism Spectrum Disorders

**DOI:** 10.3389/fnint.2018.00047

**Published:** 2018-10-04

**Authors:** Olga I. Dadalko, Brittany G. Travers

**Affiliations:** ^1^Motor and Brain Development Lab, Waisman Center, University of Wisconsin–Madison, Madison, WI, United States; ^2^Motor and Brain Development Lab, Occupational Therapy Program in the Department of Kinesiology, University of Wisconsin–Madison, Madison, WI, United States

**Keywords:** brainstem, ASD, autism, development, behavior, histology, neuroimaging

## Abstract

Autism spectrum disorder (ASD) is a neurodevelopmental condition that affects one in 59 children in the United States. Although there is a mounting body of knowledge of cortical and cerebellar contributions to ASD, our knowledge about the early developing brainstem in ASD is only beginning to accumulate. Understanding how brainstem neurotransmission is implicated in ASD is important because many of this condition’s sensory and motor symptoms are consistent with brainstem pathology. Therefore, the purpose of this review was to integrate epidemiological, behavioral, histological, neuroimaging, and animal evidence of brainstem contributions to ASD. Because ASD is a neurodevelopmental condition, we examined the available data through a lens of hierarchical brain development. The review of the literature suggests that developmental alterations of the brainstem could have potential cascading effects on cortical and cerebellar formation, ultimately leading to ASD symptoms. This view is supported by human epidemiology findings and data from animal models of ASD, showing that perturbed development of the brainstem substructures, particularly during the peak formation of the brainstem’s monoaminergic centers, may relate to ASD or ASD-like behaviors. Furthermore, we review evidence from human histology, psychophysiology, and neuroimaging suggesting that brainstem development and maturation may be atypical in ASD and may be related to key ASD symptoms, such as atypical sensorimotor features and social responsiveness. From this review there emerges the need of future research to validate early detection of the brainstem-based somatosensory and psychophysiological behaviors that emerge in infancy, and to examine the brainstem across the life span, while accounting for age. In all, there is preliminary evidence for brainstem involvement in ASD, but a better understanding of the brainstem’s role would likely pave the way for earlier diagnosis and treatment of ASD.

## Introduction

Autism spectrum disorder (ASD) is a lifelong condition affecting one in 59 children (Baio et al., 2018). This condition is clinically heterogeneous and has neurodevelopmental origin (Inui et al., 2017; Piven et al., 2017; Shen and Piven, 2017). As suggested by human genetics and studies in animal models, early neurodevelopmental alterations may give rise to diverse ASD behaviors (for a review see Lai et al., 2014; Chen et al., 2015). Specifically, autism symptoms, which include core diagnostic features of social communication deficits and restrictive, repetitive behaviors (American Psychiatric Association, 2013), as well as co-occurring sensorimotor challenges (Baranek, 1999; Fournier et al., 2010; Cascio et al., 2016), may stem from the earliest developing brain structure: the brainstem. Historically, the brainstem was at the center of the first brain-based hypothesis of ASD, which theorized that this early-developing neural structure was responsible for the behavioral features of autism (Rimland, 1964). Recently, ASD symptomatology was ascribed to the hypoplasia of the brainstem’s pons, supporting the neurodevelopmental model of autism and the critical role of the brainstem in ASD behaviors (Inui et al., 2017).

Despite these brainstem-based hypotheses, relatively few studies have investigated the brainstem in ASD limiting the ability to link the behavioral features of ASD with brainstem function. Both conceptual and methodological factors contribute to the lack of research. Conceptually, the core diagnostic features of ASD have been attributed to higher order cognitive functions, such as language (see Groen et al., 2008 for a review) and social motivation (see Chevallier et al., 2012 for a review). Likely because these cognitive domains are primarily supported by cortical brain areas, the cortex has been studied more frequently than deep brain structures such as the brainstem. Methodologically, the brainstem’s small size, functional diversity, and anatomical complexity are challenging to investigate *in vivo*. While recent technological advancements are beginning to resolve the methodological difficulties surrounding magnetic resonance imaging (MRI) of the brainstem (Ford et al., 2013; Hoch et al., 2016), the new methods have yet to be applied to the study of ASD. Although these conceptual and methodological factors may have hindered our understanding of how important the brainstem in ASD is, the few studies that have investigated the brainstem in ASD lend credence to its critical role during the neurodevelopmental unfolding of autism.

Studying how autism symptoms may stem from abnormally developing brainstem may lead to a mechanistic understanding of the neurobiological causes of ASD and may ultimately identify behaviors and biomarkers that enable earlier diagnosis. Currently, the diagnostic behavioral features of autism manifest at 24–36 months, an age by which the brainstem has undergone the majority of its maturation. The discrepancy between the ages at which behavioral diagnoses can be made and the ages at which the brainstem matures may mask a neurodevelopmental cascade originating in the brainstem that leads to core symptom appearance. Nevertheless, evidence is accumulating to implicate abnormal brainstem development in ASD. This evidence comes from diverse areas of neuroscience, spanning the fields from basic developmental biology to psychophysiology, and including the data from both humans and animal models. These widely distributed data lack a comprehensive summary. Therefore, the purpose of this review was to integrate behavioral, histological, neuroimaging, and animal evidence of brainstem contributions to ASD. Because ASD is a neurodevelopmental condition, our goal was to examine available data through a lens of hierarchical brain development, which underscores how important the guiding function of the brainstem is during neurodevelopment. For this reason, we start by discussing key developmental processes of the brainstem, and highlight behavioral features of ASD that may align with atypical development of the brainstem. We proceed by summarizing available evidence supporting the brainstem’s role in ASD, and identify the key gaps that await further research.

## Brainstem Anatomy and Development

### The Vital Complexity of the Brainstem Anatomy

**FIGURE 1:**
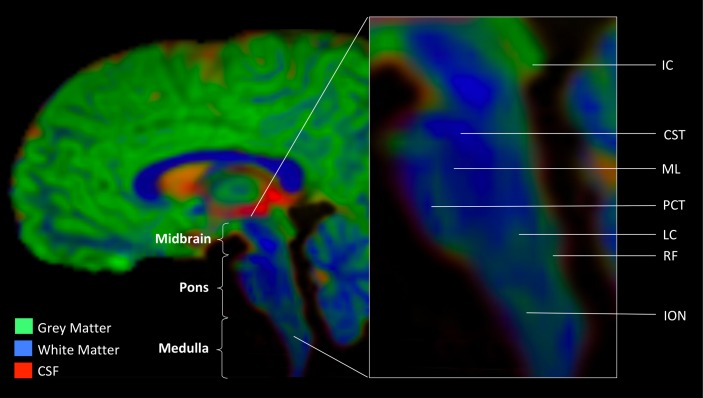
Anatomic landmarks of the brainstem. Gray matter nuclei. Midbrain: IC, inferior colliculus. Medulla: LC, locus coeruleus (noradrenergic center); RF, reticular formation (serotonergic center); ION, inferior olivary nucleus. White matter tracts. CST, cortico-spinal tract; ML, medial lemniscus; PCT, pontine crossing tract. Image was obtained from a diffusion weighted MR scan processed with MRTrix software program (mrtrix.org). Depicted is sagittal view of concatenated cerebro-spinal fluid (CSF), gray matter (GM), and white matter (WM) maps with tissue-encoded colors: red, CSF; green, GM; blue, WM.

The brainstem plays a key role in organismal survival by supporting basic physiological and behavioral functions. Diverse bodily processes such as respiration, heartbeat, stress response, gastrointestinal regulation, and basic auditory and visual functions are all supported by brainstem neurotransmission. Due to the vitality and versatility of the brainstem, this region of a vertebrate brain is highly conserved (Gilland and Baker, 1993, 2005). Perhaps because of the preservation of the brainstem’s essential role throughout evolving phylogeny, the brainstem’s organization is extraordinarily complex. Arising early in development at the closure of the neural tube, this structure does not divide into more regions like other brain structures. In contrast, the three gross anatomical regions of the brainstem (midbrain, pons, and medulla) comprise multiple substructures. The gray matter nuclei that house neuronal cell bodies are dispersed throughout intricately interwoven white matter tracts and have unique presentation, forming, for example, a gray-white matter mesh, such as the reticular formation, or a flower-like substructure, such as the inferior olivary nucleus. **Figure 1** graphically highlights the brainstem landmarks that are pertinent to the purpose of this review. Further details of brainstem neuroanatomy may be found in works specifically dedicated to this subject (for a review, see Fernández-Gil et al., 2010).

### The Guiding Role of the Brainstem in Hierarchical Brain Development

In order to explore how the brainstem may contribute to the origin of ASD symptoms, it is critical to understand the role of this brain region during development. While the core diagnostic features of autism traditionally have been ascribed to cortical atypicalities (Fletcher et al., 1995; Baron-Cohen, 2004; Ozonoff et al., 2006; Fakhoury, 2015; Hull et al., 2017; Lein et al., 2017), the cerebrum neither develops nor functions in isolation. Indeed, embryonic brain development resembles a hierarchical three-dimensional scaffold: the deep ancestral brain structures (like the brainstem) guide the formation of the evolutionarily more recent regions (like the neocortex) (Stiles and Jernigan, 2010). Additionally, cerebellar development is supported by neural signaling that originates in the brainstem’s inferior olive and pontine nuclei and innervates maturing cerebellum via mossy and climbing fibers (reviewed in Beckinghausen and Sillitoe, 2018). These processes lead to an integrative brain where both structure and function of the more complex cortical circuitry rely on the fidelity of the ancestral brain wiring and the efficacy of “lower level” processing. Even a minor disruption of the early neurodevelopmental events could lead to altered brain connectivity and function, affecting integrity of the lower level processing first, and, as a consequence, perturbing the function of the higher order neurocircuitry (Rodier, 2002; Hammock and Levitt, 2006; Thompson and Levitt, 2010; Inui et al., 2017). In all, the brainstem is uniquely positioned to be an initiating link in the unfolding of the hierarchical brain development, suggesting its implication in the whole spectrum of functions that the mammalian brain performs, including the functions traditionally attributed to higher order circuitry. Therefore, improper establishment of the brainstem wiring may ultimately result in abnormalities within phylogenetically younger brain circuitry giving rise to a spectrum of altered behaviors.

To illustrate the fundamental role of the brainstem in neurodevelopment, we highlight three key processes: (1) establishing neuroarchitecture, (2) initiating early neural activity, and (3) supporting neurocircuitry maturation. Although the mechanisms enabling these developmental processes are studied mostly in animal models, a high level of evolutionary conservation allows extrapolation of these data to form an understanding of how neurodevelopment occurs in humans (Levitt, 2003; Bystron et al., 2008; Rakic, 2009). Supporting these theoretical considerations are *in vivo* investigations in humans, suggesting that the aforementioned processes define the brain’s structural and functional connectivity (Tau and Peterson, 2009). Thus, if the brainstem indeed has a pivotal role in neurocircuitry formation, it is plausible that the brainstem may ignite a neurodevelopmental cascade leading to the atypical brain cytoarchitecture and connectivity, thereby resulting in atypical behaviors. To support this concept, we distill the most salient lines of evidence underscoring the essential role of the brainstem in neurodevelopment.

#### Neuroarchitecture Establishment

Brain neuroarchitecture begins to form during early development via such events as cell migration and differentiation, the two key processes supported by the monoaminergic circuitry residing in the brainstem (Lauder and Krebs, 1978; Whitaker-Azmitia and Azmitia, 1986; Naqui et al., 1999). Brainstem monoamines include norepinephrine, dopamine, and serotonin, which during early neurodevelopment are released in autocrine fashion to act as neurotrophins for the emerging cerebrum. Cerebral function and areal specialization are determined by an eminent milestone of neurodevelopment – cortical layering, or lamination, which is defined by the key neurodevelopmental processes of cell migration and differentiation (Rakic, 1988, 2002, 2007). Neuronal migration and differentiation in turn are supported by the brainstem’s norepinephrine, which is shown to affect how Cajal-Retzius cells orchestrate cortical layering (Naqui et al., 1999; Sarnat and Flores-Sarnat, 2002). Further, lamination is supported by the brainstem-derived serotonin, which acts as a neurotrophin and a trial neurotransmitter during early stages of neurodevelopment (Lauder and Krebs, 1978; Whitaker-Azmitia and Azmitia, 1986; reviewed in Vitalis et al., 2013). Cumulatively, evidence suggests that brainstem neurocircuitry is essential for the early neuronal migration and differentiation, positioning the brainstem at the basis of hierarchical brain development.

#### Early Neuronal Activity

Early neuronal activity that occurs before the establishment of synaptic function appears fundamental for the development of structural and functional connectivity of the brain (see Kirischuk et al., 2017 for a review). Among a few molecular processes that support pre-synaptic neuronal activity, the serotonergic system of the brainstem is one of the earliest and most critical players. Originating in the first few weeks of gestation (Sundström et al., 1993), serotonergic neurons extensively innervate the developing di- and telencephalon by the mid-gestational period (Verney et al., 2002). Then, the emerging cortical glutamatergic neurons actively take up the released serotonin using it as a trial neurotransmitter to establish initial thalamocortical connectivity (Lebrand et al., 1996). This nascent wiring is thought to serve as a blueprint for adult glutamatergic neurotransmission between the cerebrum and thalamus (Gaspar et al., 2003), supporting the imperative role of brainstem serotonergic projections in brain development. Furthermore, brainstem serotonin signaling was found to guide early neuronal activity in both the brainstem and the midbrain (Rockhill et al., 2009), which are critical for tonotopic organization of the entire auditory system, including the auditory cortex (Clause et al., 2014). The notion that the early neuronal activity in the brainstem primes the burgeoning auditory system to receive sensory-evoked signals (reviewed in Friauf and Lohmann, 1999) suggests how indispensable the brainstem may be in the neurodevelopmental cascade.

#### Neurocircuitry Maturation

Maturation of neuronal circuits involves many highly orchestrated events, one of the most critical of which is myelination. Myelination is performed by oligodendrocytes, the specialized glia cells that provide axonal insulation and enable efficient conduction of neuronal signals in the brain. Global brain myelination may actively rely on early brainstem function. At the molecular level, manipulation of the brainstem serotonergic system causes atypical oligodendrocyte function that alters brain myelination (Simpson et al., 2011; Fan et al., 2015). At the systemic level, the progression of myelination is hierarchical: the brainstem is myelinated *in utero* before the other brain regions. Importantly, the brain regions that communicate with the myelinated brainstem *in utero* are the ones to mature first as shown by their established structure and function (Mai and Ashwell, 2004). A good example of such early maturing system is the development of auditory function. The auditory brainstem matures fast, being the first brain region showing signs of myelin at 26 weeks of gestation (Moore et al., 1995). Consequently, the primary auditory cortex is the first among all cortical areas to exhibit mature lamination, myelination, and activity (Moore et al., 1995; Mai and Ashwell, 2004). Therefore, the caudo-rostral maturation of the brain’s neural circuitry further supports the fundamental role of the brainstem in neurodevelopment.

**FIGURE 2:**
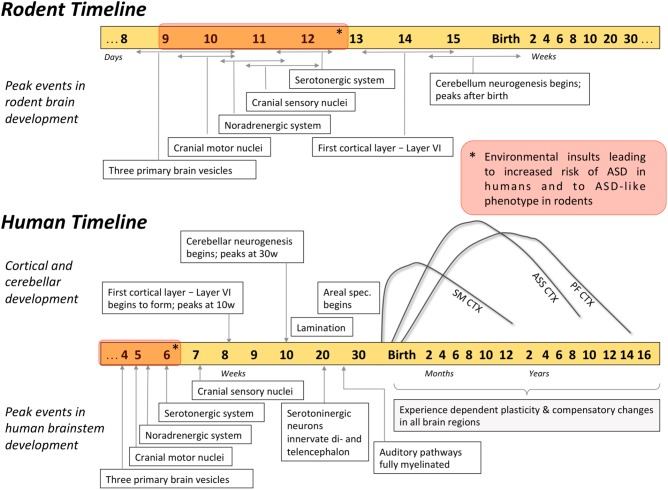
Timetable of developmental events in human and rodent brain. Development of the brainstem substructures peaks earlier compared to cortical and cerebellar regions, and can influence the development of the rest of the brain. For example, environmental insults that occur during the brainstem development (red blocks on the timelines) were associated with heightened risk of ASD. Developmental trajectories of different cortical regions highlight how malleable these brain regions are during the first months of life: SM CTX, sensorimotor cortex; ASS CTX, association cortices (parietal and temporal); PF CTX, prefrontal cortex.

Cumulatively, the evidence discussed above highlights the role of brainstem circuits that support and guide the developing brain (for schematic representation of the developmental timeline please refer to **Figure 2**). The key idea of such “bottom-up” development is that the deep, ancestral brain structures such as the brainstem may be heavily involved in pathogenesis of neurodevelopmental disorders. Although direct linkage between early brainstem development and future ASD symptoms is challenging, available evidence for neurobiological features of ASD leads us to theorize that at least some of the symptoms may be stemming from brainstem-supported neurodevelopmental events. First, atypical brainstem neurotransmission during development may have an impact on cortical lamination and areal specialization (discussed in the section “Neuroarchitecture Establishment”), resulting in abnormal cortical structure and connectivity (Olson and Walsh, 2002; Rakic, 2009). In individuals with ASD, multiple atypicalities related to cortical appearance and/or function have been previously reported (see Hardan et al., 2006 for cortical thickness; Hazlett et al., 2011, 2017 for cortical surface; Carper et al., 2016 for neurite density; Courchesne and Pierce, 2005; Müller, 2008; Minshew and Keller, 2010; Hahamy et al., 2015; Hernandez et al., 2015 for connectivity). Second, there is evidence potentially implicating brainstem serotonergic projections during early development, when serotonin plays a major role in establishing thalamocortical connectivity (discussed in the section “Early Neuronal Activity”). Specifically, serotonergic axons presented with atypical morphology in postmortem brains of children and young adults with ASD (Azmitia et al., 2011a,b), and both structural and functional thalamocortical connectivity were found to be atypical in ASD (Nair et al., 2013, 2015; Green et al., 2017; McLaughlin et al., 2017; Woodward et al., 2017). Third, it is the spontaneous activity of the brainstem (discussed in the section “Neurocircuitry Maturation”) that is thought to attune the auditory system long before the first auditory stimulus is propagated (reviewed in Friauf and Lohmann, 1999). The disruption of this developmental program may result in atypical auditory function, a behavior exhibited by many individuals with ASD (Rosenhall et al., 1999; Khalfa et al., 2004; Miron et al., 2018; Otto-Meyer et al., 2018) and associated with autism severity (Brandwein et al., 2015; Donkers et al., 2015). Overall, the principal role of the brainstem in hierarchical brain development suggests this region may have a major impact on how the symptoms of ASD unfold.

## Human Evidence for Brainstem Contributions to ASD

### Epidemiologic Data Support the Brainstem-Based Origin of ASD

The implication of atypical brainstem development in shaping the symptoms of ASD is supported by human epidemiology. According to epidemiologic findings, environmental insults that occur *in utero* (during the time of most rapid brainstem development) are associated with heightened incidence of ASD. Specifically, ASD is associated with pharmacological or immunological neurotoxicity during the first few weeks of embryonic development (**Figure 2**), particularly when the forebrain is absent and the brainstem is rapidly developing (Strömland et al., 1994; Rodier, 2002; Miller et al., 2005; for a review see Dufour-Rainfray et al., 2011). Firstly, the use of valproic acid (VPA, an anticonvulsant and a mood stabilizer) during early weeks of pregnancy was correlated with an increased incidence of ASD in children (Christensen et al., 2013). Similarly, exposure to thalidomide, an immunomodulator with anxiolytic properties, during early development increased the prevalence of ASD in Swedish population from 4:4000 to 4:86 (Strömland et al., 1994). Importantly, among various exposure times, it was the insult that occurred during rapid brainstem development (gestational age 20–24 days) that resulted in 100% of autism cases (Rodier et al., 1996). In addition, ASD occurrence that was associated with atypical prenatal maternal immune response in humans (Singer et al., 2008; Nordahl et al., 2013) was mimicked in rodents only when antibodies were introduced during gestation (Zimmerman et al., 2007; Singer et al., 2009; Martínez-Cerdeño et al., 2016), further implicating nervous system development in autism pathogenesis. Cumulatively, these epidemiological data link environmental insults sustained early *in utero* and ASD susceptibility, highlighting that environmental events linked with the neuropathology of ASD may have occurred during the period of active brainstem development.

### Early Behaviors Indicative of Atypical Brainstem Neurodevelopment in ASD

**FIGURE 3:**
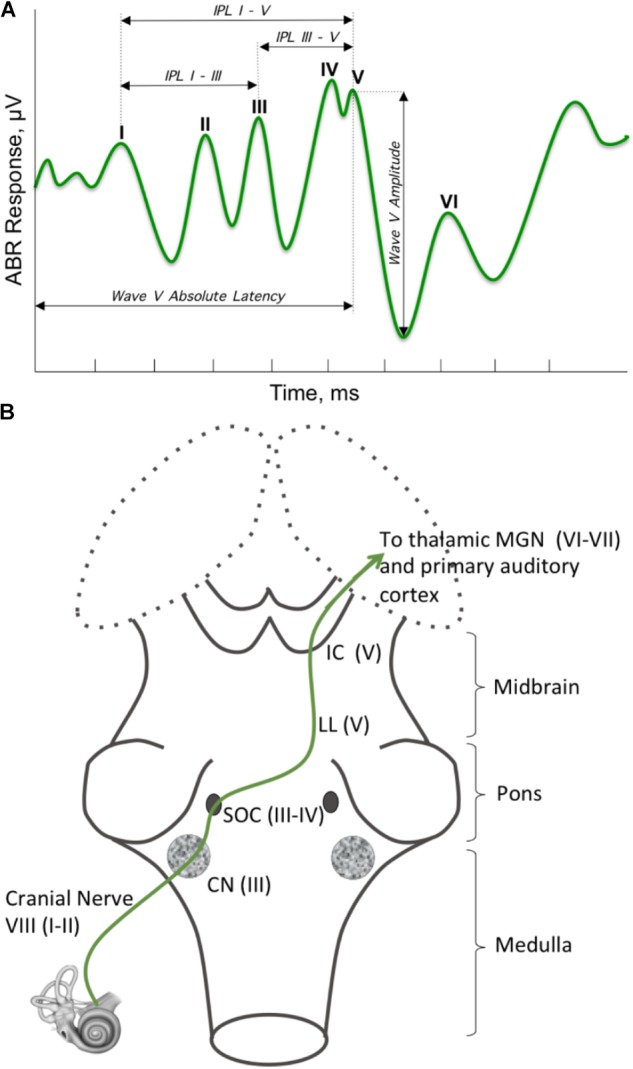
Brainstem regions supporting auditory brainstem response (ABR). **(A)** ABR is a brainstem-supported phenomenon. ABR is a series of five to seven peaks that occur in the first 10 ms following a brief stimulus, which ranges from a simple stimulus, like a click or a tone, to a more complex one, like a meaningful speech sound (/ga/). The most commonly referred to are the peaks one through five, which are labeled I–V, and evaluates the lower auditory system. **(B)** Ascending auditory signal propagates via cochlear nuclei (CN), superior olivary complex (SOC), lateral lemniscus (LL), and inferior colliculus (IC) (LL and IC are often described as the midbrain tectum), then continue to the auditory cortex through the medial geniculate body of the thalamus. Peak I originates at the distal portion of the auditory nerve (AN, cranial nerve VIII). Signal propagation through the proximal end of AN gives rise to peak II. Peak III is generated at the level of CN with participation of some regions of SOC; whereas peak IV is primarily generated by the subnuclei in the SOC. Peak V is generated by neurons within the LL and IC.

The principle of hierarchical brain development stresses the fundamental role of the brainstem, and suggests its involvement in shaping neurological symptoms. Although it is methodologically challenging to study prenatal brain development *in vivo* in humans, studies in pre-term infants can help link atypicalities in infant behavior with abnormal brainstem development. Since brainstem’s refinement and maturation occurs between 33 and 38 weeks of gestation (Darnall et al., 2006), pre-term infants may be born with an underdeveloped brainstem. Indeed, brainstem supported autonomic functions, such as arousal, temperature regulation, breathing patterns, visceral homeostasis, and heart rate variability (HRV) (Porges, 1997) were shown to be disturbed in pre-term infants (Patural et al., 2004, 2008; Longin et al., 2006). In addition to atypicalities in the autonomic nervous system, brainstem dysfunction in the perinatal period has been associated with deregulation of neurologic behaviors, such as auditory brainstem response (ABR) (Stipdonk et al., 2016). ABR’s millisecond sensitivity resolves neural responses from several brainstem substructures (**Figure 3**, for a detailed overview see Biacabe et al., 2001), thereby making ABR an excellent tool that non-invasively evaluates the brainstem function. The presence of HRV and ABR atypicalities in pre-term children supports other data showing that shorter gestational age results in a less mature brainstem (Darnall et al., 2006), leading to brainstem-based neurological dysfunctions. Given the brainstem’s role in the neurodevelopmental hierarchy, perturbed maturation of this region in pre-term infants could also make them susceptible to abnormal development of higher order brain regions, which would manifest in observable behaviors later in development.

Notably, brainstem-based neurological dysfunctions exhibited by the pre-term children were also found in children with ASD (Patural et al., 2004, 2008; Longin et al., 2006; Daluwatte et al., 2013; Bujnakova et al., 2016; Harder et al., 2016). Shared brainstem-based symptomatology suggests that ASD neuropathogenesis may stem from an atypically developing brainstem. This hypothesis is supported by the data showing that pre-term infants have higher incidence of ASD compared to the full-term children (Limperopoulos et al., 2008; Johnson et al., 2010; Pinto-Martin et al., 2011; D’Onofrio et al., 2013; Mohammed et al., 2016), with the prevalence of ASD increasing as gestational age decreases (D’Onofrio et al., 2013; Kuzniewicz et al., 2014; Darcy-Mahoney et al., 2016). Moreover, developmental trajectory of the brainstem is implicated in ASD by the abnormal progression of the brainstem-based symptoms such as HRV and ABR. Thus, aberrant HRV in neurotypically developing pre-term children normalizes by toddlerhood (De Rogalski Landrot et al., 2007), whereas HRV abnormalities in ASD persist throughout the lifespan (Daluwatte et al., 2013; Bujnakova et al., 2016; Harder et al., 2016; Kuiper et al., 2017). Such persistence of abnormal HRV in ASD suggests that the severity to which the brainstem circuitry may be altered in ASD is what leads to the atypical neurodevelopmental trajectory of this condition.

Atypical developmental trajectory of the brainstem also may lead to abnormal function of higher order brain centers in ASD. For example, abnormal ABR was associated with deficits in attention and sociability in children with ASD (Fein et al., 1981), linking aberrant brainstem neurophysiology and one of the core autism symptom domains: social communication. The link between social function and brainstem neurocircuitry is further supported by the recent findings in both typically developing children and children with ASD. For example, typically developing pre-term neonates who had abnormal ABRs also exhibited deficits in social engagement (Geva et al., 2013), which then developed into impaired social attention at 7–8 years of age (Geva et al., 2017). Concurrently, abnormal ABR of the pre-term neonates who later were diagnosed with ASD predicted the severity of the core autism symptoms such as social and linguistic competence (Cohen et al., 2013). However, ABR changes rapidly with age in ASD. According to a recent meta-analysis, an increased ABR latency in youth with ASD changes into a decreased ABR latency in adults, which is indicative of an unusually fast developmental trajectory of ABR in ASD (Miron et al., 2018). The rapid progression of ABR with age (Cohen et al., 2013; Miron et al., 2018) suggests that the neurobiology supporting ASD symptoms may be highly malleable during specific periods, opening a possibility for time-sensitive interventions. In the future, longitudinal studies investigating age-related specificity of brainstem-based behaviors in ASD may help identify vulnerable developmental stages when intervention would be able to re-wire emerging neurocircuitry.

Perturbed developmental trajectory of the brainstem in ASD may also lead to altered multisensory integration (MSI). While MSI is a complex function that involves cortical input (reviewed in Cuppini et al., 2018), its development is determined during the neonatal period by incoming auditory, visual, and somatosensory stimuli that engage the brainstem’s superior colliculus (SC) (Wallace and Stein, 1997, 2001, 2007; Burnett et al., 2004; Yu et al., 2010). Specifically, SC enables MSI by “learning” to integrate multimodal sensory input during the perinatal period (reviewed in Stein et al., 2014). From the ABR studies, perinatal period is the time when many infants who later develop ASD exhibited abnormal auditory function (Cohen et al., 2013; Miron et al., 2016). Studies in animal models showed that when the auditory function was perturbed during early infancy, it uncoupled one of the forms of MSI – audiovisual integration (Wallace and Stein, 2007). Importantly, audiovisual integration was found to be abnormal in children with ASD, whereas this deficit disappeared in adolescence and adulthood (Beker et al., 2018). Such atypical progression of audiovisual integration in ASD is especially interesting in light of the research in animals showing that compromised audiovisual integration remained in adulthood if not treated during the critical developmental period of heightened SC plasticity (Xu et al., 2017). Ability of audiovisual integration in ASD to catch up to normal levels with age suggests involvement of compensatory mechanisms that rescue this sensorimotor deficit. The neurobiology of such developmental trajectory is not well understood: these mechanisms may employ additional cortical regions, reintegrate the function of the SC, reshape the brainstem neurocircuitry supporting incoming stimuli, or use a combination of these processes. Whether compensatory mechanisms that alleviate early sensorimotor deficits occur at the cost of higher order cognitive function is unknown, but it has been theorized that audiovisual integration contributes to the core autism symptoms such as language deficits and atypical social communication (Baum et al., 2015; Stevenson et al., 2017; Beker et al., 2018). Studying neural basis of atypical progression of audiovisual integration may help us understand the developmental trajectory of the brain in ASD, thereby illuminating how early developing brain structures, such as the brainstem’s SC, contribute to the time course and presentation of ASD symptoms.

Importantly, recent work in high-risk, infant siblings of children with ASD has demonstrated behavioral features that can be detected in infancy that may align with atypicalities in brainstem neurocircuitry. In a longitudinal study of high-risk infants, children who later were diagnosed with ASD exhibited a decline in their visual attention to eyes between 2–6 months of age (Jones and Klin, 2013). Involvement of the brainstem in eye gaze direction has long been appreciated (Otero-Millan et al., 2011; Okada and Kobayashi, 2014), suggesting that this aberrant social information processing observed in 2–6 month old infants may be stemming from the atypical brainstem function. Further corroborating brainstem involvement in ASD are the data demonstrating asymmetric visual tracking in one-month old infants with ASD (Karmel et al., 2010). In addition to early eye gaze function, Karmel et al. (2010) described delayed motor performance at 7–10 months, which was in line with seminal findings showing that infants with ASD at 4–6 months of age presented with motor atypicalities (Teitelbaum et al., 1998). Since then, many lines of evidence converge to link atypical motor function in the first 24 months of life and future ASD diagnosis. Specifically, the findings revealed unusual posturing (Baranek, 1999), abnormal spontaneous movement (Phagava et al., 2008), irregular writhing and fidgeting (Einspieler et al., 2014, 2016), limited grasping skills (Libertus et al., 2014; Ekberg et al., 2016), and delay in reaching the motor milestones and/or their atypical presentation (Young et al., 2003; Sullivan et al., 2007; Ozonoff et al., 2008; Guinchat et al., 2012). With exception of a few studies (Lloyd et al., 2013; Sumner et al., 2016), accumulating findings of movement disturbances in young children with ASD suggest that an early biomarker of autism may be found within infant motor behavior domain (Zwaigenbaum et al., 2009). While in adulthood complex motor behaviors are supported by both cortical and subcortical brain regions, in infancy basic reflexes and spontaneous micro-movements are supported by the early maturing brainstem (Kobesova and Kolar, 2014). Importantly, basic reflexes (Minderaa et al., 1985; Reed, 2007) as well as spontaneous micro-movements (Brincker and Torres, 2013; Torres et al., 2013, 2017) were found to continue past infancy in ASD, corroborating brainstem involvement in life-long ASD symptoms. Moreover, in infancy and beyond, deviant motor behaviors were found to correlate with the core diagnostic features of ASD (Dziuk et al., 2007; Gernsbacher et al., 2008; Bhat et al., 2012; Radonovich et al., 2013; Hellendoorn et al., 2015; Travers et al., 2015), suggesting that the two domains may have a converging neurobiological basis.

Since motor atypicalities observed in ASD encompass a wide range of human motor function (Fournier et al., 2010) and manifest in other developmental disorders (Provost et al., 2007; Hellendoorn et al., 2015), a detailed analysis of how and when the various motor modalities overlap with the core autism features may help pinpoint early behavioral markers specific for ASD. Particularly interesting may be the inquiry into which specific motor challenges precede the core autism features, and if or how they relate to the ASD severity. Next, assessing how such infant motor behaviors are related to neonatal brain structure and function could help elucidate the neurobiological basis of ASD. If we understand which neuronal mechanisms support early motor atypicalities in ASD, we may identify malfunctioning brain regions that trigger abnormal neurodevelopmental processes leading to the core ASD symptoms observed later in life. Considering the remarkable level of neuroplasticity in infancy, a focus on finding early behavioral markers of ASD in order to intervene before the core features appear may help reroute the neuropathological development and avoid manifestation of the debilitating symptoms of ASD.

### Postmortem Histology Data Supporting Brainstem Implication in ASD

While both epidemiologic and behavioral data provide valuable insights into neurobiological basis of ASD symptoms, they can only estimate the brain neurocircuitry implicated in the condition. Histology data, on the other hand, offers direct evidence on tissue atypicalities, even though it is unable to resolve whether these brainstem tissue changes precede the manifestation of autism symptoms. Notably, only a handful of autopsy studies have investigated the brainstem in autism. This lack of research may be due to methodological constraints (i.e., difficulty obtaining adequate tissue sections), or to the specific focus of the research question. Nonetheless, the data from histological studies align with the evidence from the behavioral and psychophysiological findings stressing the brainstem involvement in ASD pathogenesis. Thus, impaired medullary arcuate nucleus (Bailey et al., 1998) may lead to the imbalance of sympathetic/parasympathetic tone and atypical HRV in ASD. Further, abnormalities found within the inferior olivary nucleus (ION) (Bauman and Kemper, 1985; Kemper and Bauman, 1993; Rodier et al., 1996; Bailey et al., 1998) could give rise to the basic motor and sensory challenges of autism. For example, eye gaze, which is often found abnormal in ASD (Leekam et al., 1998; Baron-Cohen et al., 2001) and may serve as an early biomarker of autism (Jones and Klin, 2013), is supported by the ION and the olivofloccular system (McCormick et al., 1985), both of which were found to be atypical in ASD (Bauman and Kemper, 1985; Kemper and Bauman, 1993; Rodier et al., 1996; Bailey et al., 1998; Wegiel et al., 2013). Further, superior olivary complex (SOC) was found atypical in ASD: from a near-complete absence (Rodier et al., 1996) to immature appearance (Kulesza et al., 2011). Aberrant neurotransmission in the SOC may result in such autism features as diminished ability to discriminate sound frequencies (Bouvet et al., 2016), or characteristically abnormal ABR discussed earlier (Cohen et al., 2013; Miron et al., 2018). Notably, atypical maturation of blood vessels in the brainstem was found in postmortem brains of individuals with autism (Azmitia et al., 2016). Considering how critical blood supply is for neuronal function, these data underscore other histological findings implicating the brainstem in ASD.

Diverse morphological changes found in the brainstem make it challenging to specify how particular brainstem substructures are involved in ASD. Nonetheless, the studies converge on reporting deficits unique to the brainstem. For example, in a study that evaluated the entire brain without focusing specifically on the brainstem, abnormalities in the brainstem substructures were found in all six cases with ASD (Bailey et al., 1998). Such exploratory histology is invaluable to reveal novel areas to investigate, however, it needs to be followed by a study that specifically targets the brainstem. Thus, in the exploratory study described, five out of six brains provided adequate sections to study ION; however, one out of these five appeared to have undergone severe postmortem deterioration (Bailey et al., 1998). Out of four remaining brains, three were found to have significant morphological abnormalities in the ION (Bailey et al., 1998). This analysis of results suggests that a brainstem-focused histological study could discover many missing links regarding how brainstem substructures are involved in ASD.

Another reason for diverse histological findings in the brainstem could stem from the heterogeneous nature of ASD. A number of abnormalities within the brainstem were reported on individual level, such as an aberrant white matter tract was found within the pontine tegmentum in one out of six brains (Bailey et al., 1998), or abnormal structure of both facial and superior olivary nuclei was reported in a single brain (Rodier et al., 1996). Additionally, the same brainstem substructure may present with different histological atypicalities. As such, observed abnormalities within SOC in different individuals with ASD include underdevelopment (Kulesza et al., 2011), fewer and smaller neurons (Lukose et al., 2015), aberrant geometric organization (Kulesza and Mangunay, 2008), as well as near complete absence (Rodier et al., 1996). Moreover, the contribution of age cannot be overlooked. As such, Kemper and Bauman (1993) found that somas in ION are smaller than normal in younger individuals with ASD and bigger than normal in adults with autism. Further, a large medulla and a small midbrain were found in a four-year-old child with ASD but not in the adults with ASD (Bailey et al., 1998). Overall, the evidence from the morphological findings supports the implication of the brainstem in autism. Importantly, it underscores the histological diversity of the neurobiological basis of ASD, a fact crucial to account for when we consider how heterogeneous this condition is.

### *In vivo* Neuroimaging Data

While lacking consensus in fine details, postmortem histological findings support the brainstem’s role in ASD. As for the data obtained from *in vivo* radiology studies, they lack consensus on the morphometric characteristics of the brainstem in autism. Early neuroimaging found a smaller midsagittal area of the brainstem in autism (Gaffney et al., 1988; Hashimoto et al., 1993a,b, 1995), which was in line with the data that showed increased fourth ventricle in ASD (Bauman et al., 1985; Gaffney et al., 1987). Nonetheless, others demonstrated no difference in the planimetric measure of the posterior fossa between subjects with and without ASD (Rumsey et al., 1988; Garber and Ritvo, 1992; Holttum et al., 1992; Kleiman et al., 1992). Such equivocal findings could result from the small and heterogeneous samples that were recruited for these early studies. Indeed, because ASD is characterized by its extremely heterogeneous clinical profile, it is challenging to pinpoint common neurological abnormalities in a group of participants with highly variable symptoms. Notably, substantial sample size (*N*_ASD_ = 102) (Hashimoto et al., 1995) or strict inclusion criteria for the participants with ASD (Gaffney et al., 1987, 1988; Jou et al., 2009) allowed to demonstrate altered brainstem morphometry in autism. Thus, similar to diverse tissue atypicalities found by the histology data (discussed in the section “Postmortem Histology Data Supporting Brainstem Implication in ASD”), lack of consistency among early MRI findings could be due to the vast heterogeneous profile of autism.

Group-level contrasts of the gross morphometry of the brainstem may not be enough to understand the role of this brain region in autism symptomatology. Instead, investigations into how the brainstem volume relates to heterogeneous symptoms of ASD could provide a better insight on the neurobiological basis of ASD. Thus, in a structural MRI study with substantial number of children (*N*_ASD_ = 45), a smaller brainstem volume was associated with aggression in ASD (Lundwall et al., 2017). Further, atypical *development* of the brainstem volume was reported in boys with autism (Jou et al., 2013). In this study, once again highlighting the issue of heterogeneity in autism, the individual developmental profiles were rather variable. However, this individual variability might have been more than just noise, as variability in volume was associated with individual differences in sensorimotor profiles. Specifically, brainstem-gray-matter volume related to individual differences in oral sensitivity (Jou et al., 2009), whereas brainstem-white-matter volume related to individual differences in motor performance (Hanaie et al., 2016).

In addition to gross structural volumes, assessing tissue microstructure may be able reveal important brainstem characteristics and their relation to ASD symptoms. Because the brainstem serves as a key connector for all brain-body transactions, having optimal circuit integrity within the brainstem is likely essential for effective transmission of both bottom-up and top-down neural signals. The integrity of neural circuitry can be inferred from diffusion tensor imaging (DTI), a method that estimates the underlying microstructure of the neural tissue (Beaulieu, 2002; Alexander et al., 2011). Using DTI, our previous findings demonstrated that white matter microstructure of the brainstem was related to social communication, a core ASD symptom (Travers et al., 2015). Traditionally, social communication has been attributed to higher order cortical computations (for a review see Amodio and Frith, 2006), whereas recently this view is being expanded to include lower-level processing that is thought to be integrated in social interaction brain networks (Alcalá-López et al., 2017). In line with this view, our own findings of association between the brainstem microstructure and social communication symptoms in ASD (Travers et al., 2015) raises the question of the role the brainstem plays in higher order brain function. Specifically, individuals with ASD demonstrated a significant relationship between the brainstem microstructure and grip strength, a lower-order motor function (Travers et al., 2015). Importantly, grip strength in this study also associated with social communication in ASD in such a way that that relationship became statistically insignificant once we accounted for brainstem microstructure (Travers et al., 2015). These findings suggest that the brainstem may be modulating both higher- and lower-order cognitive functions. Supporting these theoretical considerations are recent findings from the ABIDE (Autism Brain Imaging Data Exchange) dataset showing that the altered brainstem activity may be associated with the altered cortical connectivity in ASD (Vidal et al., 2017). These functional connectivity results are in line with tractography data demonstrating atypical white matter connectivity between the brainstem and both cortical (left postcentral gyrus) and subcortical (left caudate) regions in ASD (Zhang et al., 2017). Cumulatively, these data suggest that the brainstem-supported top-down and bottom-up brain-body communication may be one of the critically altered neurophysiological features of ASD.

In all, neuroimaging studies are just beginning to explore the role of the brainstem in ASD, as scanner noise can be problematic in this region and many software packages do not accommodate brainstem investigations. However, the initial results combined with current advancement in neuroimaging techniques give hope that the brainstem will receive more attention in future research. Indeed, with new techniques optimized for brainstem MRI, it has become possible to resolve many brainstem substructures *in vivo* in humans (Ford et al., 2013; Hoch et al., 2016), which will enable researchers to explore detailed brainstem microstructure and how it may relate to ASD symptoms. Moreover, with recent advancement in non-invasive imaging techniques, it is possible to evaluate the emerging brain both *in utero* and in early infancy, enabling scientists to find a possible association between perturbed brainstem function during early development and a future ASD diagnosis. Therefore, future research using these new techniques in early infancy and potentially *in utero* would enable the field to more directly test whether brainstem development is implicated in ASD.

## Animal Models of Autism in the Brainstem-Based ASD Pathogenesis

When working with human data, it is challenging to pinpoint developmental periods or molecular mechanisms that initialize neurodevelopmental cascade leading to ASD. For this reason, controlled experiments conducted in animal models can be particularly useful to propel our understanding of neurobiological basis of ASD. Due to the idiopathic nature of ASD and its multigenetic etiology (Lai et al., 2014), an all-encompassing animal model of ASD is challenging to contrive (Hyman, 2018). As a result, there are a few rodent models of ASD that can be segregated into two broad groups: (1) environmental models engineered on the basis of epidemiologic data; and (2) genetic models created by disrupting specific genes implicated in ASD. Both of these experimental designs support the construct validity of rodent models of ASD; whereas autism-like behaviors and features exhibited by these animals substantiate the models’ face validity. Animals, of course, cannot be anthropomorphized; however, rodents exhibit many human-like behaviors, mirroring anxiety, sociability, or stereotypy that can be experimentally evaluated (Silverman and Ellegood, 2018). Notably, there is variety of standardized behavioral scales to examine ASD-like behaviors when evaluating rodent models of ASD (for a review see Crawley, 2012; Kazdoba et al., 2015; Kim et al., 2016). In addition to behavioral assessment, validity of rodent models of ASD is supported by the molecular phenotypes that recapitulate human markers of ASD. For example, because elevated plasma serotonin levels was found in a third of all patients with ASD (Anderson et al., 1990; Cook and Leventhal, 1996; Hranilovic et al., 2007), hyperserotonemia in animal models of ASD is considered an autism-like feature (Narita et al., 2002). Additionally, since brain overgrowth is the prominent feature in children with ASD (Courchesne et al., 2003; Hazlett et al., 2005; Schumann et al., 2010), increased brain volume in a mouse model of ASD supports the model validity (Le Belle et al., 2014). Overall, rodent models of ASD provide a platform to investigate neurobiological basis of ASD enabling our search for vulnerable developmental periods and molecular mechanisms that can be targeted during intervention.

Aforementioned epidemiologic findings (see section “Epidemiologic Data Support the Brainstem-Based Origin of ASD”) showed that VPA/thalidomide exposure as well as maternal immune response during a particular period in pregnancy could lead to an increased risk of ASD (Strömland et al., 1994; Rodier et al., 1996; Singer et al., 2008; Nordahl et al., 2013). These human data were used to engineer environmental rodent models of ASD (Narita et al., 2002; Miller et al., 2013; Le Belle et al., 2014; Mabunga et al., 2015). Indirect evidence from the timing of engineering suggests that ASD-like behaviors in these models could stem from perturbed brainstem development (**Figure 2**). First, the insults used to engineer these rodents are introduced precisely on the gestational days when the development of the critical brainstem nuclei is at its peak (Clancy et al., 2001; Kim et al., 2011; Mabunga et al., 2015). For example, the most robust hyperserotonemia in rats was observed when the insult was introduced on gestational day nine, a time when the brainstem nuclei are actively developing (Narita et al., 2002). Moreover, for the rats to develop the most pronounced ASD-like behaviors, the critical period of VPA exposure was 12 days of gestation (Kim et al., 2011), which coincides with the peak development of the key monoaminergic brainstem nuclei (Clancy et al., 2001). In addition, *in utero* insults lead to the abnormal brainstem neurophysiology in an adult animal, linking ASD-like features and neuropathology in the brainstem. For example, ASD-like behaviors and brain overgrowth in rodents were accompanied by aberrant cytoarchitecture and altered neurotransmission in the monoaminergic centers in the brainstem (Ali and Elgoly, 2013; Miller et al., 2013; Le Belle et al., 2014). Further, *in utero* VPA exposure was found to significantly alter neuronal number (Lukose et al., 2011), as well as morphology and monoaminergic innervation of the auditory brainstem in rats (Zimmerman et al., 2018). Notably, similar histological abnormalities within the auditory brainstem were found in postmortem brains of individuals with ASD (see section “Postmortem Histology Data Supporting Brainstem Implication in ASD”; Bailey et al., 1998). Overall, these data in rodent models suggest that perturbed development of the brainstem substructures, particularly the insults during the peak formation of its monoaminergic centers, may lead to ASD-like phenotypes.

Similarly, the link between abnormal brainstem function and autism is supported by the research in genetic rodent models of ASD. These animals are engineered by recapitulating genetic mutations found in individuals with autism. Although ASD is thought to be a multigenetic disorder, single gene mutations in rodent models have proven advantageous to elucidate molecular mechanisms that lead to ASD-like behaviors. For example, autism susceptibility in humans is dramatically increased by loss-of-function mutations in neuroligin (*NLGN*) genes *NLGN3* and *NLGN4* (Jamain et al., 2003). In mice, genetic knockout (KO) of the *NL-4* (the mouse ortholog of the human *NLGN4*) leads to ASD-like behaviors such as impaired ultrasonic vocalizations (USVs) (Jamain et al., 2008), which is a behavioral proxy for human social communication (Grimsley et al., 2011; for a review see Portfors, 2007; Moy and Nadler, 2008). Importantly, the neurobiologial support for the USVs is rooted within the brainstem’s reticular formation (Wetzel et al., 1980; Yajima and Hayashi, 1983), suggesting that the ASD-like behaviors in NL-4 KO mice may be stemming from the aberrant brainstem neurotransmission. Indeed, as shown by MRI, *NL-4* KO mice have reduced brainstem volume (Jamain et al., 2008), suggesting that abnormal socialization behavior in these animals is associated with morphometric atypicalities in the brainstem. Moreover, impaired sociability was linked to the brainstem neurophysiology in a genetic mouse model of maternal 15q11-13 chromosomal triplication (Krishnan et al., 2017). Multiplication of maternally inherited 15q11-13 is a highly penetrant genetic abnormality in individuals with ASD (Glessner et al., 2009); thus recent data from a mouse model of this mutation underscores the role of the brainstem in ASD symptoms. Notably, these data linking atypical brainstem function and sociability in rodent models of autism is in line with the emerging neuroimaging data in humans (Jou et al., 2009; Travers et al., 2015).

Cumulatively, available evidence from the rodent models of ASD provides a foundation to support the brainstem involvement in ASD neuropathogenesis. However, a limited number of studies has targeted how the brainstem may be associated with ASD-like behaviors. In order to determine whether abnormal brainstem neurophysiology leads to ASD symptoms, animal model research must focus on the questions specifically targeting the brainstem. For example, existing animal models of ASD offer an uncharted territory to explore the integrity of the brainstem neurotransmission and how it may support ASD-like behaviors. Moreover, brainstem-based behaviors and autonomic functions of existing rodent models of ASD have not been evaluated and may provide valuable information about the brainstem substructures involved. Another exciting avenue for future research would be to create novel animal models of ASD by selectively manipulating the brainstem using targeted genome-editing tools such as CRISPR (Clustered Regularly Interspaced Short Palindromic Repeats) or optogenetics. The control of developmental timeframe and spatial precision allowed by these tools will permit researchers to alter specific brainstem substructures without affecting other brain regions. Such targeted manipulation will determine if atypical brainstem function indeed has a causative effect on ASD symptoms.

## Conclusion and Future Directions

The brainstem hypothesis of ASD was first proposed in the 1960s by pioneering researcher, Dr. Bernard Rimland and initiated scientific inquiry into the brain basis of ASD. Since then, many brain regions have been proposed to be the foundation of ASD symptoms. However, in pursuit of understanding the neurobiological basis of ASD, deep-brain structures like the brainstem have often been overlooked. Nonetheless, when shifting the focus from the core to the co-occurring features of ASD such as sensorimotor challenges or psychophysiological atypicalities, it becomes clear that abnormal brainstem neurotransmission contributes to ASD symptoms.

The goal of the present review was to examine what we know about the brainstem in ASD, integrating evidence from basic developmental biology to psychophysiology, and including data from both humans and animal models. While direct evidence from histological and neuroimaging findings is only starting to accumulate, epidemiological data and psychophysiology provide a foundation to support the brainstem involvement in ASD. The extent of this involvement, however, is challenging to evaluate due to heterogeneous pattern of results seen in both behavioral and neurobiological data. In order to understand whether ASD symptoms indeed stem from altered brainstem function, we can outline three major avenues for future research: (1) To account for heterogeneity of brainstem-based ASD symptoms, research may benefit from foregoing group-differences study design. Instead, we could focus on understanding neurobiological sources of individual variability in symptoms across the autism spectrum. (2) To eliminate methodological variability, we need to conduct standardized assessment of brainstem-related behaviors and link these behaviors directly to atypicalities in the brainstem using optimized psychophysiology and/or neuroimaging tools. (3) To evaluate the mechanistic role of the brainstem in ASD neuropathogenesis, we need to selectively manipulate the brainstem substructures in animal models and determine if such alterations lead to ASD-like behaviors.

**FIGURE 4:**
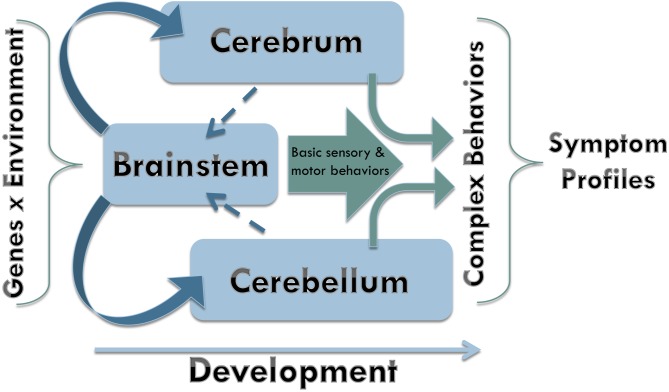
Neurodevelopmental model. Early during brain development, the brainstem exerts its major influence upon formation of both cerebrum and cerebellum. As neurodevelopment progresses, the signaling from the faster developing brainstem is receiving reciprocal signaling from the slower developing cerebrum and cerebellum. Basic sensory and motor behaviors supported by the early maturing brain structures are integrated with more complex behaviors as the rest of the brain undergoes maturation upon birth.

Based upon the findings of this review, we propose a model of brainstem influences on ASD symptoms (**Figure 4**). In essence, early perturbations affecting the developing brainstem may have cascading effects on cerebral and cerebellar development, which may lead to the expression of ASD behavioral symptoms in such a way that the more basic sensorimotor symptoms present earlier than the more complex social communication and repetitive behavior symptoms. By the time the core symptoms appear, the brain works as an integrated system that has undergone attunement to adapt to developmentally induced atypicalities. Such adaptations depend on genetic and environmental variability, leading to clinical heterogeneity commonly observed in ASD.

## Author Contributions

This manuscript was created through intensive discussions of both authors. Each author has made substantial intellectual contribution to this work, and approved it for publication.

## Conflict of Interest Statement

The authors declare that the research was conducted in the absence of any commercial or financial relationships that could be construed as a potential conflict of interest.
